# Distress financing in coping with out-of-pocket expenditure for maternity care in India

**DOI:** 10.1186/s12913-022-07656-5

**Published:** 2022-03-03

**Authors:** Shalem Balla, Md Illias Kanchan Sk, Mayanka Ambade, Babul Hossain

**Affiliations:** International Institute for Population Sciences, Mumbai, 400 088 India

**Keywords:** Maternity care, Expenditure, Distress Financing, Coping Mechanism

## Abstract

**Background:**

The cost of maternity care is seen as the barrier in utilizing maternity care, resulting in high maternal deaths. This study focuses on the distress financing and its coping mechanisms associated with maternity care expenditure in India so that corrective measures can be taken to reduce the burden of maternity care.

**Methods:**

This study used the National Sample Survey (NSS) data conducted in 20,014–15 (71^st^ round of NSS) and 2017–18(75^th^ round of NSS). We define distress financing as use of formal borrowing, borrowing from friends or family or sale of asser to finance maternity care. Percentage of pregnant/delivered females using distress financing were calculated.. The present study also used multinomial logistic regression with 95% to understand the impact of socio-economic variables on distress financing and concentration index to measure the inequality in maternity care expenditure.

**Results:**

This study found that the maternity care expenditure has decreased from the INR. 9379 in 2014–15 to INR. 7835 in 2017–18. The percentage of households using distress financing is higher among the poorest (13.2%). Almost 14% of the SC households experience distress financing. Among EAG + A states, particularly in Madhya Pradesh and Uttarakhand, the percentage of households are which experience a high level of distress financing increased from 8.9 to 18.3 and 0.7 to 8.1 from 2014–15 to 2017–18 respectively. The study finds that more urban households (37%) utilized insurance than rural households (26%). Among EAG + A states, 67.9 percent of households were dependent upon household savings, and it was 63.6 percent in the non-EAG states. The households with a high burden of maternity care expenditure were at higher risk of borrowing money to finance the cost of maternity as compared to use of savings/income for the same (relative risk (RR) (R: 2.59; *P* < 0.01; 95% CI: 2.15–3.13). Mothers belonging to the SC caste were at significantly higher risk (RR: 1.43; *P* < 0.1; 95% CI: 1.07–1.91). of using borrowings as compared to the use of income/savings. Mothers with college education were 50% more likely to use health insurance as compared to those with primary education.

**Conclusions:**

The study found that even though many programs for maternity care services are there, the maternity care expenditure, particularly the delivery care expenses, is very high in many states. The study recommends that India should increase subsidized maternity care facilities to decrease catastrophic maternity expenditure among households.

## Background

The recent estimation shows even though there is a 45% decline in global maternal mortality from 1990, preventing maternal mortality has remained a vital challenge to health systems in developing countries [1]. Around 295,000 women still die during pregnancy and childbirth, and 94% of these deaths occur in developing countries due to maternal risk factors [1]. Dating back to the 1940s, the Maternal Mortality rate (MMR) in India was 2000/100000 live births. Today, after 70 years of independence, the MMR of India has dipped to 113/100000 live births [2]. The majority of these deaths can be averted by providing proper and effective maternal care to pregnant women. A significant proportion of maternal deaths are preventable. The highest number of deaths in developing countries, including India, are due to complications like haemorrhage, eclampsia, and other avoidable causes [3]. Providing proper maternity care services could effectively reduce the number of maternal deaths [4, 5]. The cost of maternity care is seen as an access barrier to utilizing maternity care, resulting in high maternal and neonatal deaths [5, 6]. Therefore, it is a must need in countries like India to provide maternity care services free of cost. Total expenditure on health in India constitutes 3.6% of GDP, and out-of-pocket expense include 64.5% of the total health expenditure [7]. The high burden of maternal deaths and poor maternal outcomes are prognosis of low utilisation of quality health care. Studies have found a strong association between the high maternal care expenditure and low utilisation of maternal health care [8–11]. Hence, it is crucial to understand the context of high maternal care expenditure and quality of care.

The total expenditure of delivery care includes the cost for medicines and the cost for other expenses like the cost of doctor’s fee, price on diagnostic tests, transportation, etc. [9, 10]. Cost is a substantial reason for the availing of maternal care services in India. Studies have strongly suggested that a significant proportion of households are poor due to high out-of-pocket spending for health [12, 13]. The poor often use their wage income to pay off the health care expenses, wherein the less poor meet them with their savings [14]. The cost of delivery care at institutions is five times higher than at home [15]. In low-income countries, health care spending is met by direct payments, which makes them critically pervasive than the high, making them critically more pervasive than in high–income countries that use prepayment methods to finance [16]. Public facilties provide a viable solution to counter the high burden of maternity expenditure in private facilties. It has been found that there are direct and indirect expenses for maternal care where the direct expenditure for delivery care is lower than the indirect in public facilities whereas the direct costs for the same are less in private facilities than the indirect [17]. The study conducted by Mohanty, Kastor & Anshul [18] showed that women going to public health facilities increased from 11% in 2004 to 31% by 2014 while in private had increased from 12 to 20% during the same period. However, India's public health facilities are regarded as low quality because of unavailability and absence of professionals, poor infrastructure and facilities, unavailability of drugs, and proper equipment. Because of these factors, many people prefer to go to private health facilities [19–21].

According to the permanent income hypothesis, families decide current consumption patterns by assuming future income [22, 23]. If the maternity care expenditure is nominalised, households can spend from their household incomes or savings and if the households have insurances, then they will go for it. But if the maternity care expenditure is catastrophic, the families may choose to go for borrowings from banks and friends [24]. Even if the households can’t afford to pay, they will forego assets by selling to meet the expenditure [24, 25].

It is well documented that the population faces the challenges of distress financing to cope with the high levels of maternity expenditure. Distress financing means using of borrowings or the sale of physical assets [26, 27]. Distress financing will be used when households don’t have proper household savings, income, or insurances. Distress financing is limited to the poorer section of society as they have limited funding options and unavailability of economically valuable assets [28]. Previously, studies have mainly dealt with the households experiencing catastrophic maternity care expenditure [15, 17, 29, 30]. But one should emphasise understanding how the families arrange this expenditure for maternity care. Therefore, this study focuses on the coping mechanisms and distress financing in India so that corrective measures to reduce the burden of maternity care and improve maternal health can be suggested.

Previously studies have looked into the cost of maternity and analyzed the impact of conditional cash transfer schemes such as JSY on the economic burden of maternity [25, 26, 29, 30]. Studies have also looked into distress financing of institutional deliveries and maternity care. Also, studies do not provide bifurcation of costs by nature of expenditure i.e. delivery, medication, doctor consultation charges, transport etc. [25, 30]. Further, in the light of recent developments such as introduction of PMMVY (Pradhan Mantri Matru Vandana Yojana), it is imperative to generate updated estimates of distress financing due to maternity expenditure and compare it with previous estimates to track any changes thereof. Therefore, this study also looks into the level and trends of distress financing due to maternity expenditure and its change between 2014 and 2018. Since 2014 is a pre PMMVY timeline, such comparison provides us with evidence to map the impact of the policy on distress financing. We also inverstigates expenditure patterns of maternity care and its coping mechanisms for the EAG + Assam, highly experiencing maternal mortality states and non-EAG states with comparatively low maternal mortality.

## Methods

### Data source

The present study utilized the two rounds of 71st and 75th Round National Sample Survey (NSS) on the theme ‘Social consumption in India: Health to understand the household experiencing distress financing for delivery Care in India. The data from the 25th schedule of the 71st round and 75th round was used, which was completed in 2014–15 and 2017–18. The primary aim of the health survey was to collect necessary quantitative information on the health sector, the profile of ailments including their treatment, role of government and private facilities in providing healthcare, expenditure on medicines, expenditure on medical consultation and investigation, hospitalization, and expense of maternity and childbirth, etc.

The 71st round surveyed 65,932 households with 333,104 persons, and the 75th round covered a total of 1,13,823 families and 5,55,115 individuals across various States and Union Territories in India. The NSS uses a stratified two-stage design to sample census villages in the rural areas and the NSS urban frame survey blocks in the urban areas in the first stage, followed by a sampling of households in the second stage.

Data on the maternal care expenditures during the antenatal and postnatal period were collected from women aged 15–49 years who delivered their babies or were pregnant before 365 days of the survey. Information on delivery care expenditure was collected as expenses incurred during the last 365 days for in-patient medical care during childbirth. In all, 19,445 women reported to be pregnant in the 365 days before the survey; of the 14,462 women reported having a hospital birth during the reference period. So, to analyse pregnancy and maternity expenditure, we restrict our analysis to those 14,462 women who gave birth in a hospital setting in the 71st round.

In all, 32,257 women were reported being pregnant in the 365 days before the survey; of them, 27,625 women were reported having a hospital birth during the reference period and alive after delivery, so we restricted our analysis to those 27,625 women who gave birth in a hospital setting in the 75th round.

## Description of the variables

### Outcome variables:

#### Maternity care expenditure

Expenditure incurred during the last 365 days on prenatal care was considered as antenatal care expenditure. The cost incurred for the delivery as in-patient within the past 365 days was collected under separate heading. Here, the total expenses are the complete medical and non-medical expenses. Medical expenses include package, doctor's/surgeons fee, medicines, diagnostic charges, bed charges, and other costs. Along with this, transport and non-medical expenses are combined to give the total expenditures during delivery care. For postnatal care, expenditure was incurred during the last 365 days on postnatal care. The study has considered the women who have utilised the services in the past 365 days. So there are cases where women who were pregnant before one year and had delivery then but have taken PNC within the last 365 days. Correspondingly, women who were pregnant before 365 days but did not deliver but availed ANC in before last the year. The samples have been chosen based on this consideration. Assumably those who have availed ANC, have also taken PNC.

### Distress financing

It is a condition where the household meets their expenditure by borrowing and/or selling any physical assets. In this study, distress financing is any financial source other than savings and insurance. A dichotomous variable was generated with 0 as not experiencing distress financing and 1 as facing distress financing.

### Control variable

EAG + A and non-EAG states were considered as the control variable in the study. This study used EAG + A (Empowered Action Group States and Assam) as one group and non-EAG (other states) as another group for comparison purposes. EAG + A states includes Uttar Pradesh, Rajasthan, Madhya Pradesh, Chhattisgarh, Odisha, Bihar, Jharkhand, Uttarakhand and Assam. Other than these, nine states are called non-EAG states.

### Independent variables

The study used individual and household-level covariates. The basic covariates used in the analysis were the age of the mother (15–24, 25–34, 35–44 and 45 above), place of residence (rural/urban), social class (Scheduled Caste, Scheduled tribe, Others (Other backward Classes and others)), religion (Hindu, Muslim, and others), education (No literate, Literate without schooling, Primary, Secondary, Higher secondary, Graduate and above), Occupation (unpaid, student, self-employed, casual labour, pension, salaried, other) and Monthly per capita Consumption Expenditure (MPCE) quintile.

### Methodology

We calculated the total maternity care expenditure by adding all the components of maternity care (ANC, Delivery expenditure, and PNC) and divided it with household size to estimate per capita expenditure.

Total Maternity Care Expenditure (TMCE) = ANCE+ Delivery Expenditure+ PNCEH/ Household Size

$${\text{TMCE}}=\frac{ANCE+DCE+PNCE1}{HH Size}$$

where,

TMCE = Total Maternity Care Expenditure.

ANCE = Antenatal Care Expenditure.

DCE = Delivery Care Expenditure.

PNCE = Postnatal Care Expenditure.

HH Size = Household Size.

We calculated mean expenditure for maternity care expenditure and percentages for the the people experiencing catastrophic expenditure.. Inorder to make these costs comparable across timelines we have controlled for inflation using the consumer price index.

The multinomial binary logistic regression was used to analyse the impact of socio-economic variables on distress financing in the study. As with binary logistic regression, even multinomial logistic regression sets aside one category as a base category. Hence there are always C -1 parameters. Then, the logit of each non-reference category j = 1, C-12 against the reference category 0 depends on the values of the explanatory variables through:

$$\mathrm{log}(\frac{\mathrm{\pi i}\left(\mathrm{j}\right)}{\mathrm{\pi i}\left(0\right)})=\mathrm{\alpha }(\mathrm{j})+\upbeta 1(\mathrm{j})\mathrm{X}1\mathrm{i}+\cdots +\mathrm{\beta k}(\mathrm{j})\mathrm{Xki}$$

For each j = 1, C-1 where (*j*) *and* β1(*j*),…,β*k*(*j*) are unknown population parameters, π*i*(0)*and* π*i*(1) are probability parameters and k is the number of explanatory variables.

We used Stata 15.0 software for the analysis as well as the Geoda 1.16 software to make a spatial analysis of the state-level maps.

## Results

### Trends of total maternity care expenditure, delivery care expenditure and different components of average delivery care expenditure (in Rs.), India, 2014–15 to 2017–18

**Fig. 1:**
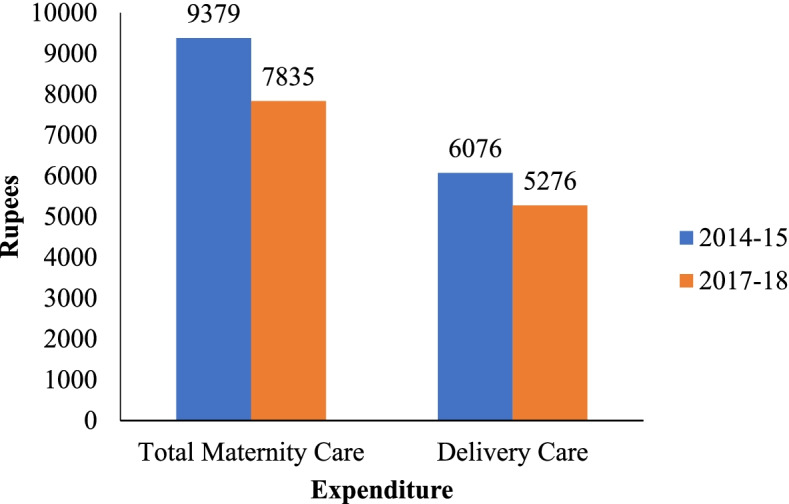
Trends of total maternity care expanditure and delivery care expenditure, India, 2014–17

**Fig. 2:**
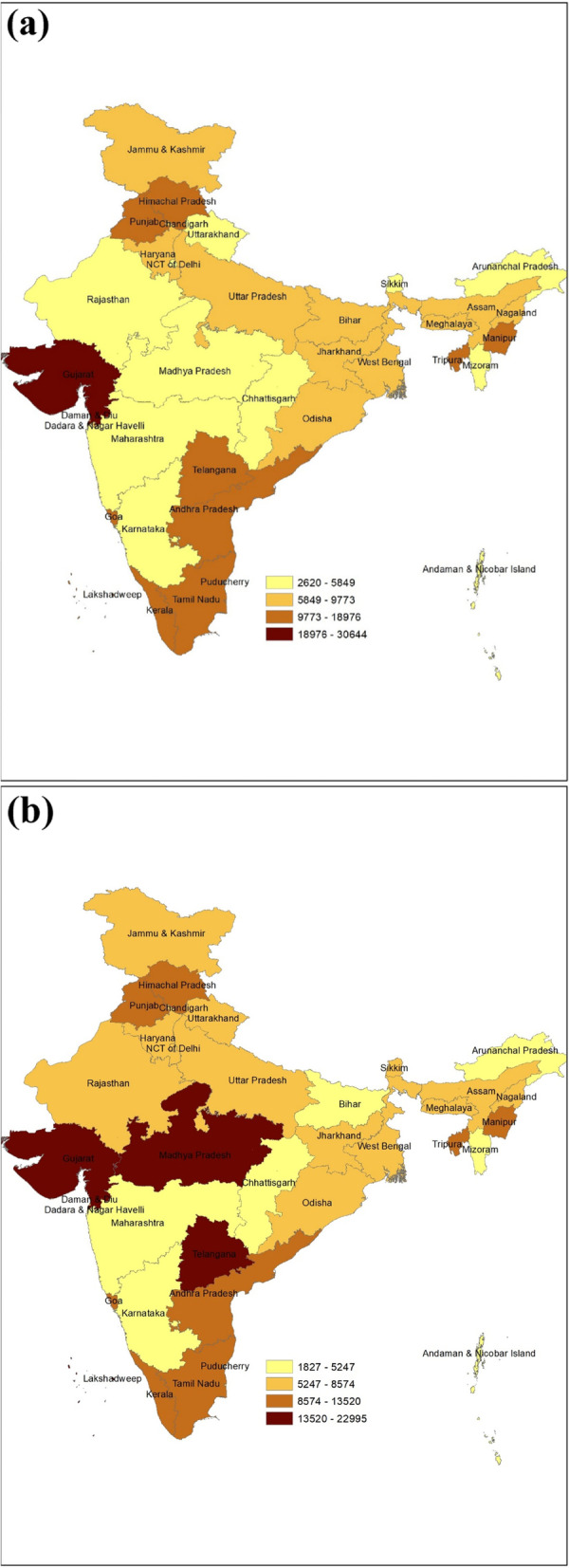
**a** State wise total maternity care expenditure, 2014–15, India. **b** State wise total maternity care expenditure, 2017–18, India

Figure 1 shows the average total maternity care and delivery care expenditures were Rs. 6076 and Rs. 5276 respectively in 2017–18. However, delivery care expenditure consisted lion's share (almost 90%) of maternity care expenditure in India. Figures 2a and b shows the average total maternity care expenditure by Indian states from 2014–15 to 2017–18. The average expenditure remained high among the developed states like Andhra Pradesh, Telangana, Tamil Nadu, Goa, Gujarat and Punjab in the both time periods. But in the states like Madhya Pradesh, Uttarakhand, Manipur the expenditure has increased form the period 2014–15 to 2017–18.

It is also found that the majority of delivery care expenditures were comprising of package components, doctor's/ surgeon's fee, and spending on medicines (Table 1). Further, there was not much difference in component-wise average delivery care expenditure from 2014–15 to 2017–18, except for the package component in India.

**Table 1:** Components of Average Delivery Care Expenditure (in INR.), India, 2014–15 to 2017–18

**Variables**	EAG + A		Non-EAG		India	
	**2014–15**	**2017–18**	**2014–15**	**2017–18**	**2014–15**	**2017–18**
Package Component	-	1291	38,353	3622	38,353	2366
Doctor's/ Surgeon's fee	812	895	2498	2476	1669	1624
Expenditure on Medicines	1286	1414	387	2187	1733	1770
Expenditure on Diagnostic Tests	426	502	60	1081	662	769
Expenditure on Bed Charges	389	423	418	1049	720	712
Other medical expenditures	332	361	628	880	533	601
Expenditure on Transport	465	451	1014	584	494	512
Other Non-medical Expenditure	717	725	1238	1162	878	926
Total	4180	3491	8009	7362	6076	5276

*Note*: There is no samples for package component in EAG states for the period 2014–15

### Trends of distress financing for maternity care expenditure

Table 2 depicts the percentage of households using distress financing with different selected socio-economic characteristics during 2014–15 to 2017–18. Results show that in India, distress financing decreased from 17% in 2014–15 to 12% in 2017–18. However, the percentage of households using distress financing in the 45 + age group increased from 7% in 2014–15 to 28% in 2017–18. Among wealth quartile, the percentage was low among 4^th^ quartile (9%). The percentage of SC households (14.1%) experiencing distress financing was comparatively high compared to other categories in 2017–18.

**Table 2:** Percentage of households using distress financing by selected socio-economic variables and EAG + A and non-EAG states, India,2014–15 to 2017–18

Variables	Categories	2014–15	2017–18
**Age of the mother (in Years)**	15–24	19.5	14.3
	25–34	15.0	11.4
	35–44	13.3	8.8
	45 +	6.6	28.0
**Residence**	Rural	17.7	12.7
	Urban	15.2	11.7
**Literacy of mother**	No literate	19.4	13.7
	Literate without schooling	23.9	6.9
	Primary	16.8	11.7
	Secondary	17.7	13.2
	Higher secondary	16.4	12.3
	Graduate and above	10.6	10.3
**Occupation of the mother**	Unpaid	-	12.4
	Student	-	8.6
	Self-employed	-	14.6
	Casual labour	-	18.2
	Pension	-	8.5
	Other	-	18.7
	Salaried	-	9.3
**Caste**	ST	12.3	10.9
	SC	19.1	14.1
	Others	17.1	12.1
**Religion**	Hindu	17.1	12.8
	Muslim	18.0	11.6
	Others	12.3	9.6
**Wealth quartile**	Quartile 1	18.4	13.2
	Quartile 2	19.3	13.8
	Quartile 3	17.1	13.2
	Quartile 4	13.4	9.5
**Type of health facility**	Public	13.3	11.2
	Private	23.6	15.4
**EAG + A**	Uttarakhand	0.7	8.1
	Rajasthan	7.8	7.5
	Uttar Pradesh	14.2	14.3
	Bihar	25.1	7.7
	Assam	3.3	3.6
	Jharkhand	13.4	15.0
	Odisha	22.4	6.5
	Chhattisgarh	13.7	6.8
	Madhya Pradesh	8.9	18.3
	**EAG + A**	**14.0**	**11.3**
**Non-EAG**	**Non-EAG**	**20.0**	**13.8**
**Total**	**India**	**17.0**	**12.5**

*Note*: The number of samples for the sale of physical assets category are very less to calculate averages

**Fig. 3:**
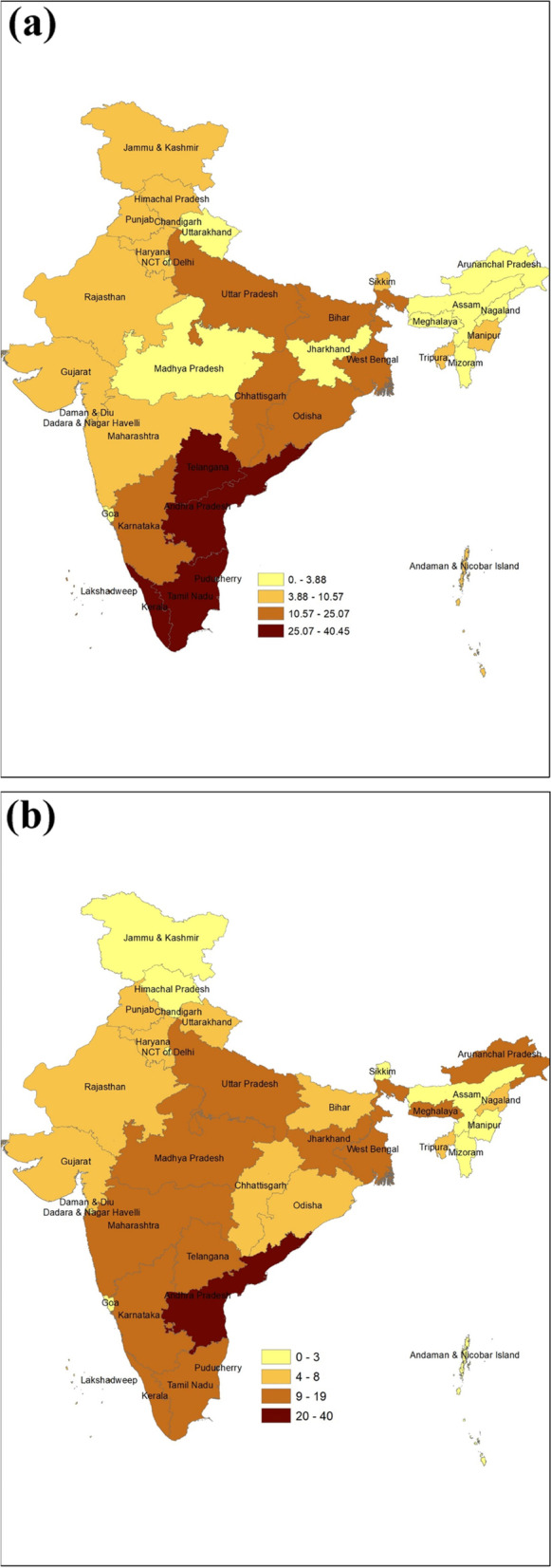
**a** Percentage of People Experiencing Distress Financing for Maternity Care Expenditure, 2014–15, India. **b** Percentage of People Experiencing Distress Financing for Maternity Care Expenditure, India, 2017–18

The study also finds that 11% of the population belonging to EAG + A states incurred distress financing in 2017–18. Among the EAG + A states, the percentage of households facing distress financing was highest in Bihar (25.1%) in 2014–15 and it decreased to 7.7% in 2017–18. Only in Madhya Pradesh, the percentage of families facing distress financing increased (8.9% in 2014–15 to 18.3% in 2017–18), and Madhya Pradesh had the highest rate of households experiencing distress financing in 2017–18. Along with Madhya Pradesh, the households from Uttar Pradesh, Madhya Pradesh, Jharkhand, Tamil Nadu, Andhra Pradesh, Telangana, and Karnataka were also experiencing higher distress financing (Fig. 3).

### Coping mechanism for the total maternity care expenditure

Table 3 depicts the incidence of using various sources of finance as a coping mechanism by households to meet the total maternity care expenditure in India during 2017–18. More than half of the households (58%) utilized savings as a coping mechanism to meet maternity care expenditure in India. In contrast, about one-third of households used insurance for the same. Financial support from friends and relatives, and the sale of assets, contribute towards almost 8% of the total maternity care expenditure.

**Table 3:** Incidence (%) of Use of Different Sources of Finance as a Coping Mechanism to meet the Total Maternity care by Households in India, 2017–18

Variables	Categories	Savings/House Hold Income	Borrowings	Insurance	Others
**Age of the Mother (in Years)**	15–24	61.4	3.8	27.3	6.8
	25–34	61.1	3.1	30.6	4.8
	35–44	62.5	3.2	30.3	3.4
	45 +	46.8	2.1	30.4	20.7
**Residence**	Rural	63.6	3.7	26.4	5.9
	Urban	54.6	2.7	37.4	4.8
**Literacy of mother**	No literate	64.7	3.6	23.9	7.5
	Literate without schooling	80.0	2.1	15.6	2.3
	Primary	63.8	3.0	26.8	5.5
	Secondary	61.8	3.8	28.0	5.9
	Higher secondary	59.9	3.4	31.6	5.0
	Graduate and above	51.5	2.8	42.2	3.2
**Occupation of the mother**	Unpaid	61.6	3.4	29.1	5.5
	Student	65.3	0.1	27.1	7.6
	Self employed	61.0	4.1	26.2	8.8
	Casual labour	61.7	3.8	22.6	10.6
	Pension	75.7	0.0	16.9	7.4
	Other	52.1	10.3	34.2	3.0
	Salaried	39.9	3.2	54.3	2.4
**Caste**	ST	59.0	2.2	33.5	5.0
	SC	63.2	4.3	25.7	6.4
	Others	61.0	3.3	29.8	5.4
**Religion**	Hindu	61.5	3.6	28.8	5.7
	Muslim	62.1	3.0	28.4	5.9
	Others	54.0	2.2	40.8	3.0
**Wealth Quartile**	Quartile 1	66.6	3.8	22.4	6.9
	Quartile 2	64.3	3.9	24.7	6.7
	Quartile 3	60.2	3.8	29.8	5.6
	Quartile 4	53.8	2.2	40.4	3.1
**Type of Health Facility**	Public	64.85	2.0	26.56	6.5
	Private	53.95	6.8	35.82	3.4
**EAG + A**	Uttarakhand	72.1	2.9	20.2	4.9
	Rajasthan	40.2	3.5	54.6	0.8
	Uttar Pradesh	74.8	3.8	12.1	8.7
	Bihar	74.4	4.5	19.1	2.1
	Assam	76.3	2.4	20.6	0.8
	Jharkhand	49.5	2.7	41.8	5.5
	Odisha	64.4	2.3	31.4	2.0
	Chhattisgarh	79.9	2.0	14.3	3.3
	Madhya Pradesh	72.5	2.4	9.8	14.6
	**EAG + A**	**67.9**	**3.4**	**22.3**	**5.9**
**Non-EAG**	**Non-EAG**	**53.6**	**3.5**	**37.3**	**5.3**
	**India**	**57.8**	**3.1**	**34.4**	**4.4**

*Note*: The number of samples for the sale of physical assets category are very less to calculate averages

We found that more than 64% of households from rural areas used savings to meet the maternity care expenditure, while 55% of the urban population did so. However, more urban households (37%) utilized insurance than rural households (26%). As far as the caste of the respondents was concerned, the highest percentage of SC households utilized household savings than other caste people. Nevertheless, the percentage of households using insurance was higher among the non-SC/ST category. Among the wealth quartiles, 40 percent of households who were in 4^th^ quartile utilized insurance, and 53 percentage of households used borrowings, whereas 66 percent of households in quartile 1 used household savings, and 22 percentage households used insurance as the coping mechanism to meet maternity care expenditure. Fifty-four percent salaried mothers used insurance, and 39% of households were more dependent on family savings. Eighty percent of households used household savings as a coping strategy in which the women were literate without schooling. The percentage of households having insurance increased with education. Across religious groups, households from Muslim and Hindu communities almost equally (62%) used household income or savings as a coping mechanism in maternity care.

Among EAG + A states, 67.9 percent households were dependent upon household savings and it was 63.6 percentage in non-EAG states. The proportion of households that met their expenditure through borrowings was almost the same in both EAG + A and non-EAG states (3.4% and 3.5% respectively). Nevertheless, the scenario reversed itself in the case of insurance. The proportion of mothers who used insurance in EAG + A states (22%) was less than non- EAG states (37%). Among the EAG + A states, a low percentage of households had savings in Jharkhand (49.5%) and Rajasthan (40.2%) to bear the maternity care expenditure. In the case of insurances, it was low in Madhya Pradesh and highest in Rajasthan (54.6%).

### Determinants of using distress financing as a coping mechanism for maternity care expenditure

Table 4 Multinomial Logistic Regression shows the determinants of the type of coping mechanisms among households. Relative risk is the probability of an event occurring in the exposed group versus the probability of the event occurring in the non-exposed group. It gives the relative risk ratio of use of different sources of finance with reference to Savings.

**Table 4:** Multinomial Logistic Regression showing Association between Coping Mechanism and Selected Socio-economic Variables

**Coping Mechanisms**		**Borrowings**		**Insurance**		**Other Coping Mechanisms**	
	**Variables**	**RRR**	**95% Conf. Interval**	**RRR**	**95% Conf. Interval**	**RRR**	**95% Conf. Interval**
**CMCE**	CMCE (no) ®						
	CMCE (yes)	2.59***	(2.15, 3.13)	1.39***	(1.28, 1.52)	1.27*	(1.02, 1.57)
**Residence**	Rural®						
	Urban	0.72***	(0.62, 0.85)	1.11***	(1.05, 1.18)	0.99	(0.87, 1.14)
**Literacy of mother**	No literate®						
	Literate without schooling	0.44	(0.16, 1.22)	0.73	(0.51, 1.04)	0.64	(0.32, 1.27)
	Primary	0.83	(0.66, 1.06)	1.21***	(1.09, 1.34)	0.83	(0.68, 1.02)
	Secondary	0.65***	(0.53, 0.81)	1.29***	(1.17, 1.41)	0.95	(0.79, 1.13)
	Higher secondary	0.53***	(0.41, 0.69)	1.35***	(1.21, 1.5)	0.78*	(0.62, 0.98)
	Graduate and above	0.38***	(0.29, 0.51)	1.81***	(1.62, 2.02)	0.75*	(0.58, 0.97)
**Wealth quartile**	Quartile 1®						
	Quartile 2	1.22	(0.98, 1.5)	1.1*	(1.01, 1.2)	1	(0.85, 1.18)
	Quartile 3	1.24*	(1.01, 1.53)	1.31***	(1.2, 1.42)	0.86	(0.73, 1.02)
	Quartile 4	1.07	(0.84, 1.37)	1.74***	(1.59, 1.9)	0.63***	(0.51, 0.77)
**Caste of the mother**	ST®						
	SC	1.43*	(1.07, 1.91)	0.51***	(0.46, 0.56)	1.23	(0.99, 1.53)
	Others	1.12	(0.86, 1.46)	0.51***	(0.48, 0.56)	1.19	(0.98, 1.45)
**Type of health facility**	Govt/Public Hospital®						
	Private Hospitals	3.07***	(2.56, 3.68)	1.05	(0.98, 1.13)	0.82*	(0.7, 0.98)
	Constant	0.04***	(0.03, 0.05)	0.54***	(0.48, 0.6)	0.09***	(0.07, 0.11)

*Note*: The number of samples for the sale of physical assets category are very less for regression analysis. ® = Reference category, *P* < 0.1 = *, *P* < 0.04 = **, *P* < 0.01 = *** Level of significance for multinomial logistic regression

The households with a high burden of maternity care expenditure were at significantly higher risk (RR: 2.59; *P* < 0.01; 95% CI: 2.15–3.13) of using borrowings as compared to Income/Savings to meet the maternity care expenditure. The mothers using private health facilities were more likely to use borrowings (RR: 3.07; *P* < 0.01; 95% CI: 2.56–3.68) than the mothers using public health facilities. Urban households were at lower risk of using borrowings (0.72; *P* < 0.01; 95% CI: 0.62–0.85) compared to income/savings with rural households. Urban households were at a higher chance of using insurance (RR: 1.11; *P* < 0.01; 95% CI: 1.05–1.18) as compared to income/savings than their rural counterparts. The mothers belonging to the SC caste were at significantly higher risk (RR: 1.43; *P* < 0.1; 95% CI: 1.07–1.91) of using borrowings compared to the use of income/savings than their counterparts. There was a low risk of dependent upon borrowings compared to income and savings when the mothers were educated secondary and above. The educated mothers were more likely to use insurance (Primary = 1.21; *P* < 0.01; 95% CI: 1.09–1.34; Secondary = 1.29; *P* < 0.01; 95% CI: 1.17–1.41; Higher secondary = 1.35; *P* < 0.01; 95% CI: 1.21–1.5; Graduate and above = 1.81; *P* < 0.01; 95% CI: 1.62–2.02) than the women who had no education or who were in the literate without schooling group as compared to income and savings.The households with a high burden of maternity care expenditure were at significantly higher risk (RRR: 1.39; *P* < 0.01; 95% CI:1.28–1.52) of using insurance as compared to the use of Income/Savings to meet the maternity care expenditure. The mothers belonged to 2^nd^, 3^rd^ and 4^th^ quartiles were more likely to use insurance (quartile 2 = 1.1; *P* < 0.1; 95% CI: 1.01–1.2; quartile 3 = 1.31; *P* < 0.01; 95% CI: 1.2–1.42; quartile 4 = 1.74; *P* < 0.01; 95% CI: 1.59–1.9) than the 1^st^ quartile mothers as compared to income and savings.

The households with a high burden of maternity care expenditure were at significantly higher risk (RRR: 1.27; *P* < 0.1; 95% CI:1.02–1.57) of using other coping mechanisms as compared to the use of Income/Savings. The households belonged to Quantile 4 (rich) category were at significantly lower risk (RRR: 0.63; *P* < 0.01;0.95% CI:0.51- 0.77) of using other coping mechanisms as compared to the use of Income/Savings to cope with the households belonged to other three categories. The maternal who took treatment in a private health facility were at significantly lower risk (RRR: 0.82; *P* < 0.1;95% CI:0.7- 0.98) of using other coping mechanisms as compared to the use of Income/Savings to cope with the maternal who took treatment in the public health facility.

**Fig. 4:**
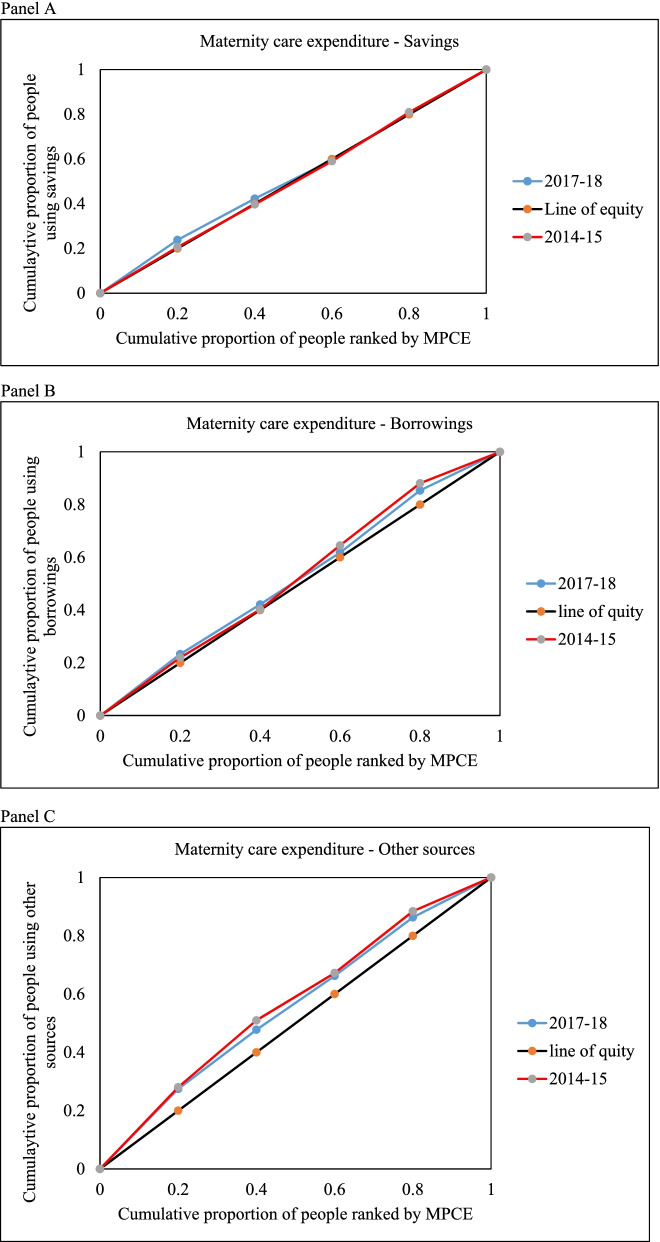
Concentration curves for savings, borrowings and other sources

Figure 4 shows the concentration curves (CCs) of the cumulative proportion of households ranked by MPCE on the x-axis upon the cumulative proportions of households using the different coping mechanisms for maternity care expenditure. The concentration curves for savings, borrowings, and other sources show the level of inequality among different quantile. A higher concentration of savings is observed among the wealthier group, whereas poorer households are experiencing a higher concentration of borrowing and other sources. On the other hand, the incidence of distress financing follows a socio-economic gradient where economically weaker sections have a higher risk of financial hardship in maternity care. The comparison between the slope of the CC during 2014–15 to 2017–18 shows the incidence of other sources continues to the pro-rich. At the same time, the slope of the CC for borrowings has moved closer toward the line of equality, demonstrating that the incidence is less concentrated among economically weaker sections in 2017–18 than in 2014–15.

## Discussion

Over the last few decades, the world has witnessed a significant improvement in health in general and maternal health in particular [31]. The rise in health expenditure coincided with this improvement [32]. It is usually presumed that increase in health expenditure will automatically improve the health outcome. Countries like India still struggle to prevent maternal mortality and improve maternal health at a promising level [33, 34]. In particular, the EAG states are experiencing a massive burden of health care expenditure, particularly the maternity care expenditure [31]. Increasing the utilization of Maternity care is one of the aims of the National Health Mission (NHM) and others to reduce maternal mortality, neonatal mortality and decrease catastrophic spending on maternal care [18]. This study provides the situational analysis for maternity care expenditure and throws light on how the population meets their distress financing in post NHM periods (2014–15 & 2017–18).

Results from this study depicted that, the catastrophic maternity care expenditure has decreased from the period 2014–15 to 2017–18 [18, 30]. In this study, we found that doctor’s and medical fees are the main components in the expenditure, which plays an important role in increasing the expenditure and the households fall into a catastrophic trap [30]. of the reason might be poor health expenditure and fewer doctors in the country [35]. India has a much lower proportion doctor population ratio than the WHO norm [36].

The percentage of households using distress financing is higher among the poorest section. But, still, there is a high percentage of households using distress financing among wealthy people. This may be because of the high aspiration of people to hospitals that have better health facilities [25, 27]. It is also documented in several previous research that the health transition is much faster than the socio-economic transition in the country [3, 37], and that leads to usage of distress financing even among rich households.

The study also highlighted that people from the SC caste are experiencing high distress financing. Similar finding observed in other previous studies [38, 39] depict that because of lack of health knowledge, low education, and low economic status among the SC communities [40–42]. Similarly, when we analysed the regional variation, EAG + A states, particularly Madhya Pradesh, is experiencing a high level of distress financing [8]. We also found that in developed states like Andhra Pradesh and Telangana a high percentage of households experiencing distress financing. This may be because of the aspiration of better health care access, leading to high health care expenditure [12, 23]. The out-of-pocket expenditure on delivery care in developing countries like African countries Kenya, Tanzania is much lower. Still, it is higher in developed countries like United States, Poland, Israel, Switzerland, and Russia. This is because they provide highly subsidized maternity health care facilities in comparison with India [43, 44]. The percentage of households going for treatment in private hospitals are using higher levels of borrowings than the public hospital utilizing patients [25, 27]. The expenditure in public hospitals is lower than the private hospitals. But, due to the poor health infrastructure in public hospitals, people prefer private hospitals even though they will fall in the catastrophic expenditure trap [35].

This study also shows how people are using different sources of finance as coping mechanisms to meet maternity care expenditure. The study found that around sixty percent of households are using household savings to meet the maternity expenditure. Similar finding is also observed in the previous studies [30, 39]. The percentage of households using insurance in rural areas is less than the urban households. The lack of awareness about the benefit of insurance coverage is responsible for the same [45, 46]. The literate households are using savings as a coping strategy than their counterpart. Studies have documented that the knowledge to save and awareness about schemes is much higher among literate households than the less educated households [17, 30]. We also found that borrowings are the primary source used to cope with distress financing in all states [8, 25]. Urban households are at a lower risk of using borrowing as a coping mechanism than rural households. Previous studies also documented that rural households have a massive burden for borrowing money to cope with health care expenses [27, 47]. Rural households have low health insurance, and struggling to arrange daily expenses are significant factors [9].

The maternal using private health facilities were more likely to use borrowings than the mothers using public health facilities because the cost of treatment is high comparing to public hospitals [39]. Urban maternals were at lower risk of using borrowings compared to income/savings with rural households. The women belonging to the SC caste were at significantly higher risk of using borrowings compared to the use of income/savings than their counterparts because of lack of health knowledge, low education and low economic status among the SC communities [40–42]. There was a low risk of dependent upon borrowings compared to income and savings when the mothers were educated secondary and above. As expected the educated mothers were more likely to use insurance than the women who had no education or who were in the literate without schooling group as compared to income and savings.

The Government of India announced the Pradhan Mantri Matru Vandana Yojana (PMMVY) in January 2017, which provided partial compensation or wage loss in the form of cash support of INR 5000 to pregnant and nursing women. A sum of INR 1000 is given upon early pregnancy registration at an Anganwadi Centre (AWC) / Approved Health Facility, a sum of INR 2000 after 6 months of pregnancy on receiving at least one ante-natal check-up (ANC), and a sum of INR 2000 after child birth is registered and the child has received the first cycle of Bacillus Calmette Guerin (BCG), Oral Polio Vaccine (OPV) and Diphtheria vaccines. The programme aims to improve pregnant women's and breastfeeding mothers' health-seeking behaviour. However the high codt of maternity care imperiments the success of this programme, our findings show a slight reduction in maternity care expenditure and distress financing indicating the positive impact of PMMVY [48]. Despite the reduction, the current mean expediture for maternity care and its related distress financing are high. It is higher among SC, poor, less educated maternals and rural households. Better improvement of PMMVY among these vulnerable groups will instrument further reduction in materternity cost. For the same we also suggest further enquiries into pitfalls of PMMVY implementation process.

## Conclusions

A high level of out-of-pocket expenditure (OOPE) is indicative of a regressive health care system. There have been several policies and programs that aim at the betterment of maternity care service utilisation, wherein the ultimate aim of all the programs like JSY is to decrease maternal mortality rate by providing health services in the needed times remarkably increasing the institutional delivery for which the policies and programs offer cash incentives and thereby increasing the utilisation and to reduce burden among the household. The study found that even though many programs for maternity care services are there, the maternity care expenditure, particularly the delivery care expenses, is very high in many states. Obviously India should increase subsidized maternity care facilities to decrease catastrophic maternity expenditure among households. The study also highlighted that the population prefers private hospitals for delivery care instead of higher expenses at private hospitals. Therefore, it can be suggested that improving public health facilities for maternity care are required to reduce the catastrophic maternity care expenditure on households that may finally reduce the economic burden. Cost of delivery plays a central role in increasing the total maternity care expenditure. So, better health facilities are needed in local healthcare centers, leading to the low cost delivery. The doctor's fee is the main component in delivery care expenditure. The proportion of doctor's fees is high in comparing with other components. The doctor’s fee is nominal in public health facilities, but the fee is high in private sectors. So, implementation of policy to maintain the doctor’s fee standard in all health facilities should be regulated. Insurance coverage for maternity care expenditure is highly recommended for all the women in the reproductive age group to lower catastrophic maternity care expenditure and out-of-pocket expenditure in the country.

### Limitations

This study has some limitations. First, the expenditure data is self-reported and not verified from other sources. Second, recall bias in reporting the actual expenditure for doctor’s fee, medicines expenditure, diagnostic tests expenditure, etc. Third, the study only included direct costs. Finally, in most cases, the expenditures associated with the family members or the persons who accompanied the patients during hospital admission and transportation are not included in the study.

## Data Availability

The study is based on secondary data analysis. No data was collected for this study. The data are available for free on the NSSO website (http://mospi.nic.in/unit-level-data-report-nss-75th-round-july-2017-june-2018-schedule-250social-consumption-health).

## Abbreviations

ANC: Antenatal Care
EAG + A: Empowered Action Group + Assam
MMR: Maternal Mortality Rate
MPCE: Monthly Percapita Consumption Expenditure
NRHM: National Rural Health Mission
NSSO: National Sample Survey Organization
OBC: Other Backward Class
OOPE: Out-of-pocket Expenditure
PNC: Postnatal Care
SC: Scheduled Caste
ST: Scheduled Tribe
WHO: World Health Organization
